# Active site plasticity and possible modes of chemical inhibition of the human DNA deaminase APOBEC3B

**DOI:** 10.1096/fba.2019-00068

**Published:** 2019-12-24

**Authors:** Ke Shi, Özlem Demir, Michael A. Carpenter, Surajit Banerjee, Daniel A. Harki, Rommie E. Amaro, Reuben S. Harris, Hideki Aihara

**Affiliations:** ^1^ Department of Biochemistry, Molecular Biology and Biophysics University of Minnesota Minneapolis MN USA; ^2^ Masonic Cancer Center University of Minnesota Minneapolis MN USA; ^3^ Institute for Molecular Virology University of Minnesota Minneapolis MN USA; ^4^ Department of Chemistry and Biochemistry University of California, San Diego La Jolla CA USA; ^5^ Center for Genome Engineering University of Minnesota Minneapolis MN USA; ^6^ Howard Hughes Medical Institute University of Minnesota Minneapolis MN USA; ^7^ Northeastern Collaborative Access Team Cornell University Lemont IL USA; ^8^ Department of Medicinal Chemistry University of Minnesota Minneapolis MN USA

**Keywords:** active site plasticity, APOBEC3B, cytosine deaminase, DNA mutation, enzymes, molecular dynamics simulations, protein structure, small molecule, x‐ray crystallography, zinc ion

## Abstract

The single‐stranded DNA cytosine deaminase APOBEC3B (A3B) functions in innate immunity against viruses, but it is also strongly implicated in eliciting mutations in cancer genomes. Because of the critical role of A3B in promoting virus and tumor evolution, small molecule inhibitors are desirable. However, there is no reported structure for any of the APOBEC3‐family enzymes in complex with a small molecule bound in the active site, which hampers the development of small molecules targeting A3B. Here we report high‐resolution structures of an active A3B catalytic domain chimera with loop 7 residues exchanged with those from the corresponding region of APOBEC3G (A3G). The structures reveal novel open conformations lacking the catalytically essential zinc ion, with the highly conserved active site residues extensively rearranged. These inactive conformations are stabilized by 2‐pyrimidone or an iodide ion bound in the active site. Molecular dynamics simulations corroborate the remarkable plasticity of the engineered active site and identify key interactions that stabilize the native A3B active site. These data provide insights into A3B active site dynamics and suggest possible modes of its inhibition by small molecules, which would aid in rational design of selective A3B inhibitors for constraining virus and tumor evolution.

## 1 INTRODUCTION

The hallmark activity of the APOBEC family of enzymes is catalyzing a zinc ion‐mediated hydrolytic deamination of 2'‐deoxycytidine to 2'‐deoxyuridine in single‐stranded (ss)DNA [reviewed by Refs. 1, 2]. In addition to AID and APOBEC1, human cells have the potential to express up to seven different APOBEC3 (A3) enzymes (A3A‐D, A3F‐H). These enzymes have overlapping functions in providing innate immunity to viral infections with, for instance, A3D/F/G/H capable of restricting the replication of the retrovirus HIV‐1. Although best characterized for roles in restricting retrovirus and retrotransposon replication (with obligate ssDNA intermediates), several family members have also been implicated in the restriction of small and large double‐stranded (ds)DNA tumor viruses. For instance, A3B and A3A have been implicated in mutagenesis of papillomaviruses and polyomaviruses^3^, ^4^ and, most recently, A3B in restricting and mutagenizing Epstein‐Barr virus and Kaposi's sarcoma herpesvirus (related gamma‐herpesviruses).^5^ Although susceptible viruses encode measures to counteract restriction by A3 enzymes, strong cases can be made for each virus leveraging A3 mutagenic potential to promote its own evolution (ex. immune escape). Thus, there is an impetus to develop small molecule inhibitors of A3 enzymatic activity as a means of constraining virus evolution and preventing adverse outcomes such as drug resistance.^1^, ^6^

The antibody gene diversification enzyme AID and several antiviral A3 enzymes have also been implicated in genomic DNA mutations in cancer (reviewed by Refs. 7, 8). In particular, A3B is the leading candidate for solid tumor mutagenesis because it is the only constitutively nuclear family member, overexpressed in many different cancer types, active in cancer cell line extracts, and upregulated by cancer‐causing viruses (HPV, above). High A3B levels in tumors have also been correlated with poor clinical outcomes, including drug resistance and metastasis.^10^, ^11^ A3A and A3H have also been implicated in solid tumor mutagenesis,^12^, ^13^, ^14^ but the endogenous forms of these enzymes have thus far proven challenging to detect and study in model systems such as tumor‐derived cell lines.

A striking feature of A3B mutagenesis in cancer is that the resulting mutation spectrum can be reconciled structurally. The APOBEC mutation signature in cancer is defined as C‐to‐T and C‐to‐G base substitution mutations within 5'‐TC(A/T) trinucleotide motifs (reviewed by Refs. 7, 8, 15, 16). 5′‐TCG trinucleotides are also preferred substrates for A3B/A‐catalyzed deamination^17^, ^18^, ^19^ but this motif complicates bioinformatic analyses due to overlap with methyl‐CG motifs, which are prone to spontaneous deamination and associate with the biological age of cancer patients (ie, aging mutation signature). Relative to the target cytosine base (0 position), the 5′ nucleobase (−1 position) is particularly important. In both A3B catalytic domain (A3Bctd*, see below) and A3A x‐ray structures with bound ssDNA substrates, the −1 T forms three hydrogen bonds with loop 7 residues, two direct and one water‐mediated.^19^, ^20^ Other nucleobases, particularly purines, clash with loop 7 residues and are poorly accommodated. Moreover, the integrity of the atomic structures has been validated by systematically changing a single residue (Asp314 in A3Bctd and Asp131 in A3A) to Glu, which alters the hydrogen bonding potential and changes the enzyme's intrinsic preference from 5′‐TC to 5′‐CC.^19^

Given the importance of A3B in cancer, small molecule inhibitors of A3B are desirable. However, the lack of A3B‐small molecule co‐structures constrains such efforts; particularly, structure‐guided A3B ligand discovery and optimization. Here we report high‐resolution structures of an A3B chimera, one in the presence of a small molecule, as well as dynamics of a catalytically competent A3B construct with an engineered active site, in order to address this knowledge gap and help expedite the rational design of selective A3B inhibitors.

## 2 MATERIALS AND METHODS

### 2.1 Crystal structure determination

A3Bctd‐QMΔloop3‐GL7 (A3B‐GL7), which has a substitution of A3G residues 317 to 322 (DQGRCQ) for residues 315 to 320 (YDPLYK) of A3Bctd‐QMΔloop3, was expressed in *Escherichia coli* strain C41(DE3)pLysS and purified using nickel‐affinity and size‐exclusion chromatography as reported.^21^ Zinc was not included in buffers used during the protein purification or crystallization. Protein concentrations were determined based on UV absorbance, using theoretical extinction coefficients calculated from the amino acid sequence of each protein. QM and Δloop3 stand for a set of solubility‐enhancing mutations, F200S/W228S/L230K/F308K, and replacement of the loop 3 region spanning Ala242 to Tyr250 by single serine, respectively. The crystal bound to 2‐pyrimidone (PDB ID: 6nfl) was obtained by the hanging drop vapor diffusion method, by mixing purified A3B‐GL7 protein in 20 mmol/L Tris‐HCl (pH 7.4), 0.5 mol/L NaCl, 5 mmol/L β‐mercaptoethanol and 0.4 mol/L neutralized 2‐pyrimidone (2‐hydroxypyrimidine)‐hydrochloride with a well solution consisting of 0.1 mol/L Bis‐Tris‐HCl (pH 5.5) and 25% w/v PEG3350. The apo crystal (PDB ID: 6nfm) was obtained similarly but in the presence of 10 mmol/L α/β‐2'‐deoxyzebularine instead of 2‐pyrimidone, and a well solution consisting of 0.2 mol/L LiCl, 20% w/v PEG6000, and 0.1 mol/L HEPES‐NaOH (pH 7.0). We did not observe electron density for 2'‐deoxyzebularine. The iodide‐bound crystal (PDB ID: 6nfk) was obtained using a E255Q mutant of A3B‐GL7 in the presence of a single‐stranded oligo DNA containing the CCC target motif, using a well solution consisting of 45 mmol/L NaI and 8.6% w/v PEG3350. The crystal did not contain DNA. All x‐ray diffraction data were collected at the Northeastern Collaborative Access Team beamlines (24‐ID‐C/E) at the Advanced Photon Source, Argonne National Laboratory, and were processed using XDS.^22^ The structures were determined by molecular replacement phasing using PHASER,^23^ using the A3Bctd‐QMΔloop3 structure as the search model. Model building was performed using COOT^24^ and the structures were refined using PHENIX.^25^ While we do not observe electron density for hydrogen atoms at 1.7 Å resolution, we chose the 2‐pyrimidone tautomer over 2‐hydroxypyrimidine to describe the bound ligand in the 6nfl structure, based on hydrogen bonding potentials with surrounding protein groups. The summary of x‐ray data collection and model refinement statistics, along with PDB accession codes for the atomic coordinates and structure factors, is shown in Table 1.

**Table 1:** Data collection and model refinement statistics

PDB ID	Iodide‐bound	2‐pyrimidone‐bound	Apo
	6nfk	6nfl	6nfm
**Data collection**			
Resolution range (Å)	35.8 ‐ 1.86 (1.93 ‐ 1.86)	41.9 ‐ 1.73 (1.80 ‐ 1.73)	37.4 ‐ 2.5 (2.6 ‐ 2.5)
Space group	P4_1_2_1_2	P4_1_2_1_2	P4_1_2_1_2
Unit cell			
*a,b,c* (Å)	50.6 50.6 149.3	50.7 50.7 149.3	50.3 50.3 149.8
Total reflections	110 026 (10 511)	129 728 (12 773)	51 953 (5231)
Unique reflections	17 023 (1631)	21 113 (2077)	6968 (674)
Multiplicity	6.5 (6.4)	6.1 (6.1)	7.5 (7.8)
Completeness (%)	99.37 (98.43)	99.82 (99.95)	99.91 (100.00)
*I/σ(I)*	14.0 (1.4)	16.1 (2.1)	10.9 (1.2)
R‐merge (%)	5.74 (130)	7.36 (85.1)	18.05 (189)
R‐meas (%)	6.24 (141)	8.06 (93.1)	19.41 (202)
R‐pim (%)	2.42 (54.0)	3.23 (37.2)	7.04 (72.2)
CC_1/2_	0.999 (0.704)	0.999 (0.689)	0.996 (0.500)
**Refinement**			
Reflections	17 023 (1630)	21 123 (2077)	6966 (674)
# for R‐free	873 (87)	1074 (101)	325 (34)
R‐work (%)	21.14 (30.21)	15.99 (24.45)	22.4 (33.0)
R‐free (%)	25.69 (42.98)	19.27 (27.16)	28.0 (33.7)
# non‐H atoms	1615	1713	1533
Macromolecules	1526	1546	1510
Ligands	13	31	1
Solvent	76	136	22
Protein residues	182	182	183
r.m.s.deviation			
Bond lengths (Å)	0.013	0.019	0.019
Bond angles (º)	1.08	1.20	0.44
Ramachandran plot			
Favored (%)	97.78	98.33	97.24
Allowed (%)	2.22	1.67	2.76
Outliers (%)	0.00	0.00	0.00
Average B‐factor	48.24	27.29	54.14
Macromolecules	48.07	26.35	54.26
Ligands	57.67	44.46	60.25
Solvent	50.08	34.13	46.04

Note

Statistics for the highest resolution shell are shown in parentheses.

### 2.2 Molecular dynamics simulations

Molecular dynamics simulations were performed with Amber18 program^26^ using Amber FF14SB force field^27^ for amino acids, the Cationic Dummy Atom Model (https://doi.org/10.1007/s008940050119) for the catalytic zinc ion and the zinc‐coordinating residues and TIP3P model for water molecules. The starting coordinates of the A3B_wt_‐GL7 were generated by modeling in the loop7 of A3G onto the wild‐type A3B structure. For the loop7 of A3G to graft, we used residues 313‐324 (A3G numbering) of chain A in PDB ID: 3ir2. For the A3B structure, we used the wild‐type A3B model we generated from PDB ID: 5cqi by molecular modeling previously.^21^ The A3B_wt_‐GL7 system was prepared by Leap solvating in a water box with 10 Angstrom buffer, then neutralizing with 7 Na^+^ ions and finally obtaining a salt concentration of 0.2 M by adding more Na^+^ and Cl^‐^ ions. The final system consisted of 35,151 atoms. The system went through multi‐step energy minimization, followed by gradual heating and multi‐step equilibration with decreasing restraints. The detailed MD protocol used for this system is exactly the same as outlined in our recent publication.^28^ The final production MD trajectories with three independent copies, 500 ns each, were generated in an NPT ensemble at 310 K without any restraints. For each MD copy, 10,000 data frames are saved and analyzed. The RMSD plots depicting the stability of MD runs are shown in Figure 6C.

### 2.3 In vitro enzyme activity assays

The single‐stranded DNA cytosine deaminase activities of A3Bctd‐QMΔloop3 and A3B‐GL7 were measured by incubating 800 nmol/L of the optimal oligonucleotide substrate for each protein at 37°C for 2 hours with 100 μmol/L of each enzyme in 25 mmol/L HEPES pH 7.4, 50 mmol/L sodium chloride (Figure S1), or 50 μmol/L of each enzyme in 25 mmol/L HEPES pH 7.4, 150 mmol/L sodium chloride, 0.1% Triton X‐100 (Figure S2). To test inhibition of both enzymes by iodide, either KI or NH_4_I was added at indicated concentrations. NH_4_I did not affect the pH of the buffer up to 200 mmol/L. To test inhibition of A3B‐GL7 by 2‐hydroxypyrimidine, 0 to 400 mmol/L of 2‐hydroxypyrimidine hydrochloride, which had been neutralized with an equimolar amount of NaOH, was included in the reaction. We also performed a titration of sodium chloride from 150 to 550 mmol/L to separately test the effect of salt. These reactions were followed by a 5 minute incubation at 95°C to inactivate A3B, then 10 minutes at 37°C with 0.1 units of UDG (NEB, Figure S1) or 1 hour at 37°C with 0.5 units of UDG (Figure S2). In parallel, UDG was tested against a uracil containing oligo under the same conditions as in the corresponding A3B reactions to control for differences in salt concentration and to determine if 2‐hydroxypyrimidine or iodide was affecting uracil removal. Following uracil removal, all reactions were treated with 100 mmol/L sodium hydroxide at 95°C for 5 minutes. 1 pmol of each treated oligo was separated by 15% TBE‐Urea PAGE. Gels were imaged with a Typhoon FLA‐7000 (GE).

### 2.4 Zinc content analysis

The zinc content of purified A3Bctd‐QMΔloop3 and A3B‐GL7 was analyzed using a zinc quantification kit (Abcam) according to the manufacturer's instructions. The proteins were first denatured by the addition of an equal volume of 7% trichloroacetic acid. Following an incubation at room temperature for 15 minutes, the proteins were pelleted by centrifugation and supernatant was subjected to the colorimetric assay. Absorbance at 560 nm was measured on a Tecan Spark 10M plate reader.

## 3 RESULTS

### 3.1 Design of A3B‐GL7

**Figure 1:**
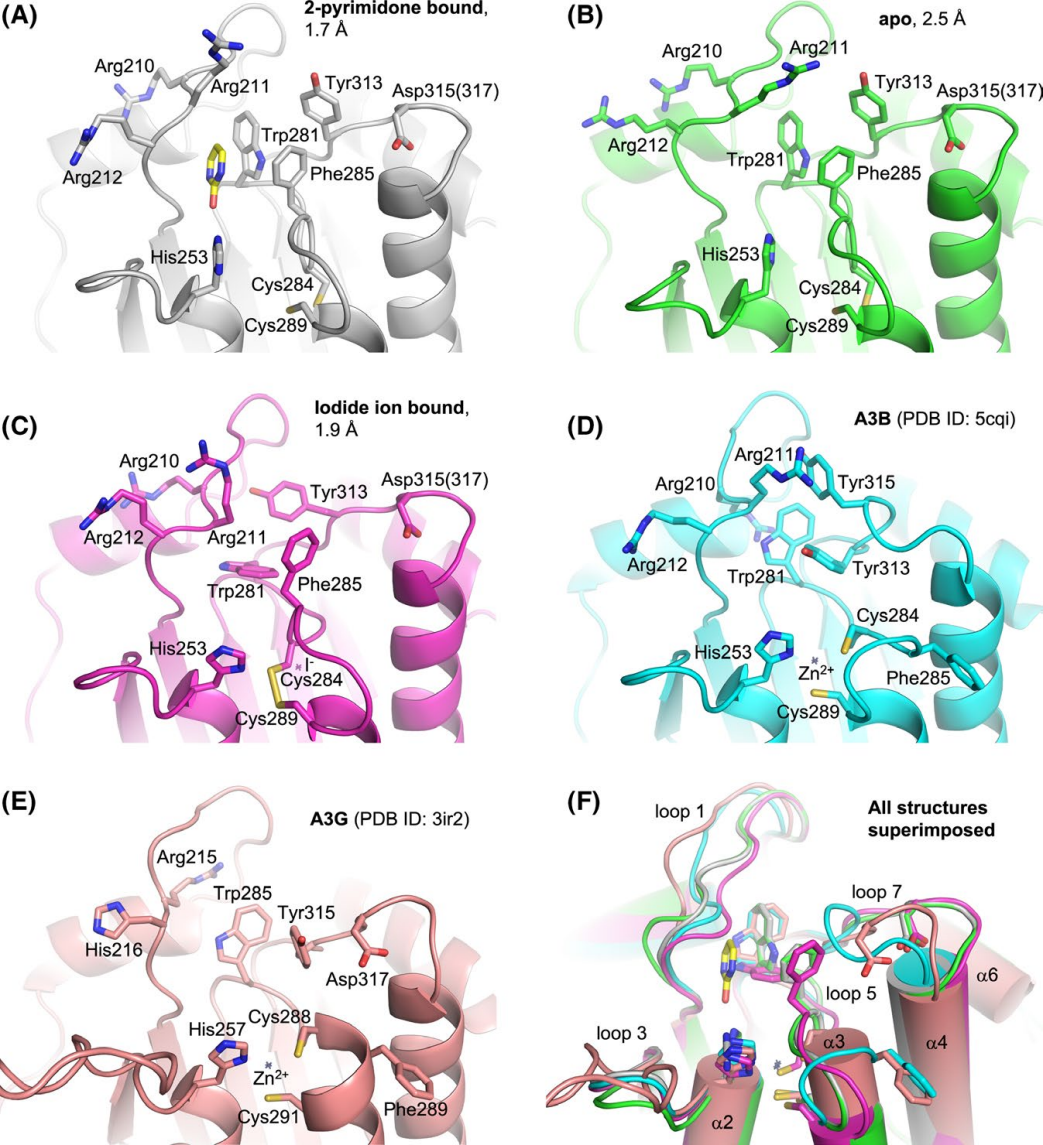
Active site comparisons. Structures around the active site of A3B‐GL7 determined in this study (A‐C), compared to those of previously reported A3Bctd structures^21^ (D) and A3Gctd structures^44^ (E). A superposition of all these structures is shown in (F). Key side chains are shown as sticks. Asp315 in A3B‐GL7 is labeled according to A3B and corresponds to Asp317 in A3G loop 7. Small crosses mark the positioning of zinc or iodide. 2‐pyrimidone bound in the active site of A3B‐GL7 (A) is shown as sticks (yellow: carbon; red: oxygen; blue: nitrogen)

We previously reported structures of the A3B C‐terminal catalytic domain (A3Bctd), determined in several different crystal forms. All DNA‐free A3Bctd structures have thus far shown a tightly closed active site, in which loops 1 and 7 on either side of the ssDNA‐binding groove associate with each other and block access of ssDNA substrates^21^, ^29^ (Figure 1). Replacing loop 1 with that from A3A, a Z1‐class cytosine deaminase >90% identical to A3Bctd, facilitated structural studies of ssDNA‐bound A3B (A3Bctd*)^19^ by freeing‐up loop 7 residues for direct interactions with ssDNA substrate, as well as by introducing a histidine into loop 1 that helps to hold ssDNA in the active site. We therefore reasoned that, replacing A3Bctd loop 7 with that from A3G catalytic domain, another Z1 cytosine deaminase, may similarly facilitate opening of the active site to allow binding of small molecules. Thus, in this study, we used A3Bctd‐QMΔloop3‐GL7 (hereafter called A3B‐GL7), a highly soluble and catalytically active A3Bctd construct with a grafted loop 7 derived from A3G (Figures S1 and S2). Loop 7 residues are critical determinants of the local intrinsic sequence preferences for deamination of each A3 enzyme with, for instance, A3B and A3A preferring 5′‐TC substrates and A3G preferring 5′‐CC substrates.^17^, ^18^, ^30^, ^31^, ^32^ This intrinsic preference is explained at the atomic level by two direct and one water‐mediated hydrogen bonds between A3B/A loop 7 residues and the −1 T^19^, ^20^ and three direct hydrogen‐bonds between A3G loop 7 and the −1 C.^33^ Consistent with these structural observations, the chimeric protein A3B‐GL7 was shown to preferentially deaminate cytosine in a 5′‐CC target sequence in DNA, similarly to wild‐type A3G.^21^

### 3.2 A3B‐GL7 crystal structure shows a zinc‐free active site

**Figure 2:**
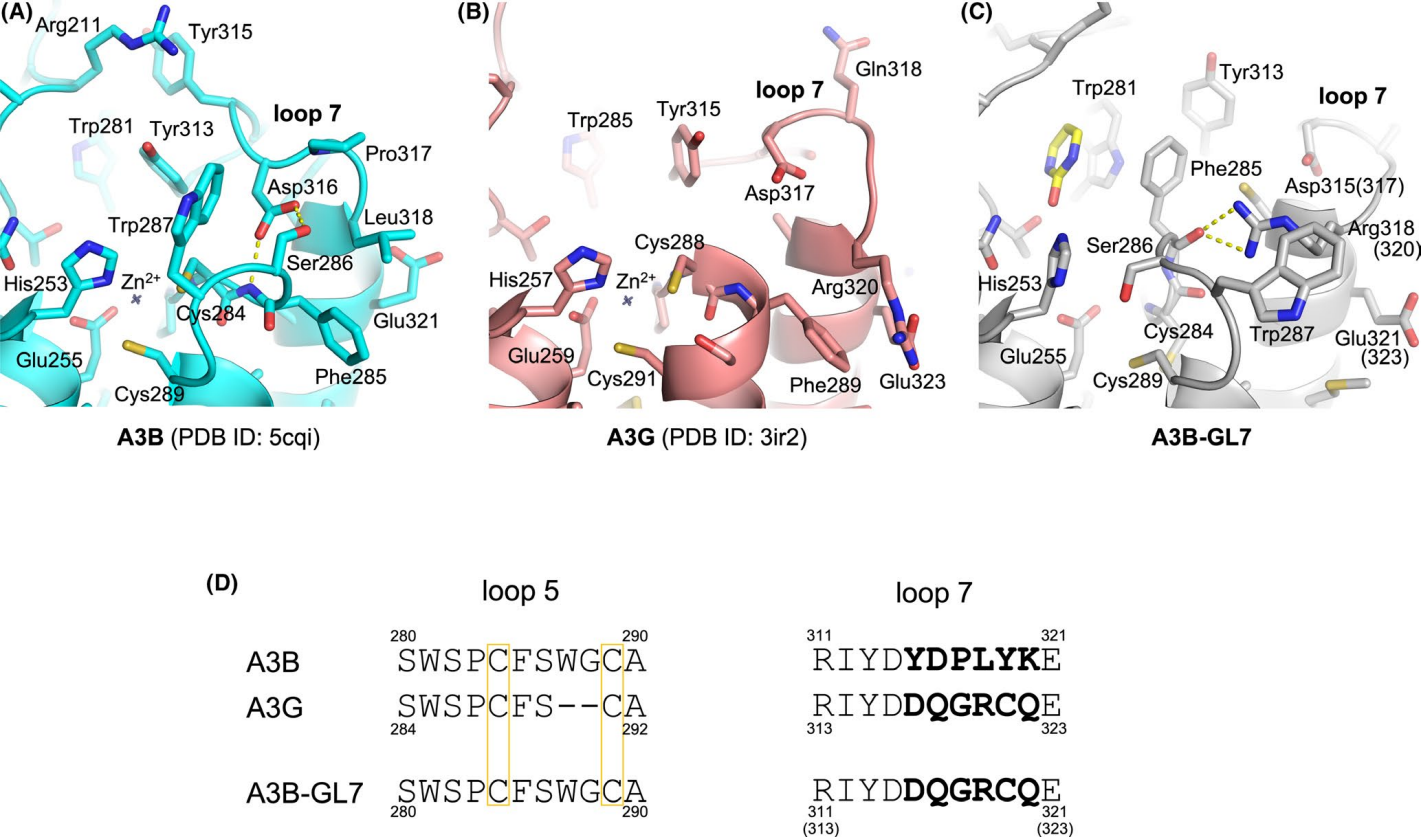
Distinct conformations of loops 5 and 7 from A3B and A3G. Active site conformations as observed in crystal structures of A3Bctd^21^ (A), A3Gctd^44^ (B), and 2‐pyrimidone‐bound A3B‐GL7 (C), with the side chains of all residues shown as sticks. Key hydrogen bonds between loops 5 and 7 are indicated by yellow dotted lines. (D) Amino acid sequence alignment for loops 5 and 7. The zinc‐coordinating cysteine residues are boxed. The loop 7 residues that differ between A3B and A3G are in bold

We first co‐crystallized A3B‐GL7 with 2‐pyrimidone (2‐hydroxypyrimidine), which had been used previously as a mechanism‐based inhibitor for zinc‐dependent cytosine deaminases,^34^, ^35^ and determined its structure at 1.7 Å resolution (Figures 1A and 2C). The refined structure of A3B‐GL7 showed an overall fold very similar to those of prior A3Bctd structures^21^, ^29^ (Figures 1D and 2A), except that loops 1, 5, and 7 that shape the active site pocket adopt distinct conformations (Figure 1F). Notably, the electron density clearly indicated that the catalytically essential zinc ion is absent and, accordingly, the zinc‐coordinating residues (His253/Cys284/Cys289) have been rearranged. A zinc‐free active site was reported previously for a variant of the A3F catalytic domain, where lower pH promoted reversible dissociation of zinc.^36^ As our 2‐pyrimidone‐bound A3B‐GL7 crystal was obtained in an acidic condition with pH 5.5, we considered the possibility that loss of the zinc ion was due to low pH or alternatively the very high concentration (0.4 mol/L) of 2‐pyrimidone, which may chelate zinc.^37^ Thus, we next crystallized A3B‐GL7 in a neutral condition at pH 7.0 in the presence of 10 mmol/L of 2′‐deoxyzebularine, a nucleoside analogue bearing 2‐pyrimidone as the nucleobase,^38^ and determined its structure at 2.5 Å resolution. This structure, unexpectedly, still showed a zinc‐free active site similar to that described above, but without a ligand (apo form; Figure 1B). These results combined to indicate that A3B‐GL7 is prone to losing zinc even in a neutral pH condition. This inference was further supported by the significantly lower zinc content of purified A3B‐GL7 compared to that of its parental construct A3Bctd‐QMΔloop3 without the loop 7 replacement (Figure S3).

### 3.3 Extensive rearrangement observed in the A3B‐GL7 active site

**Figure 3:**
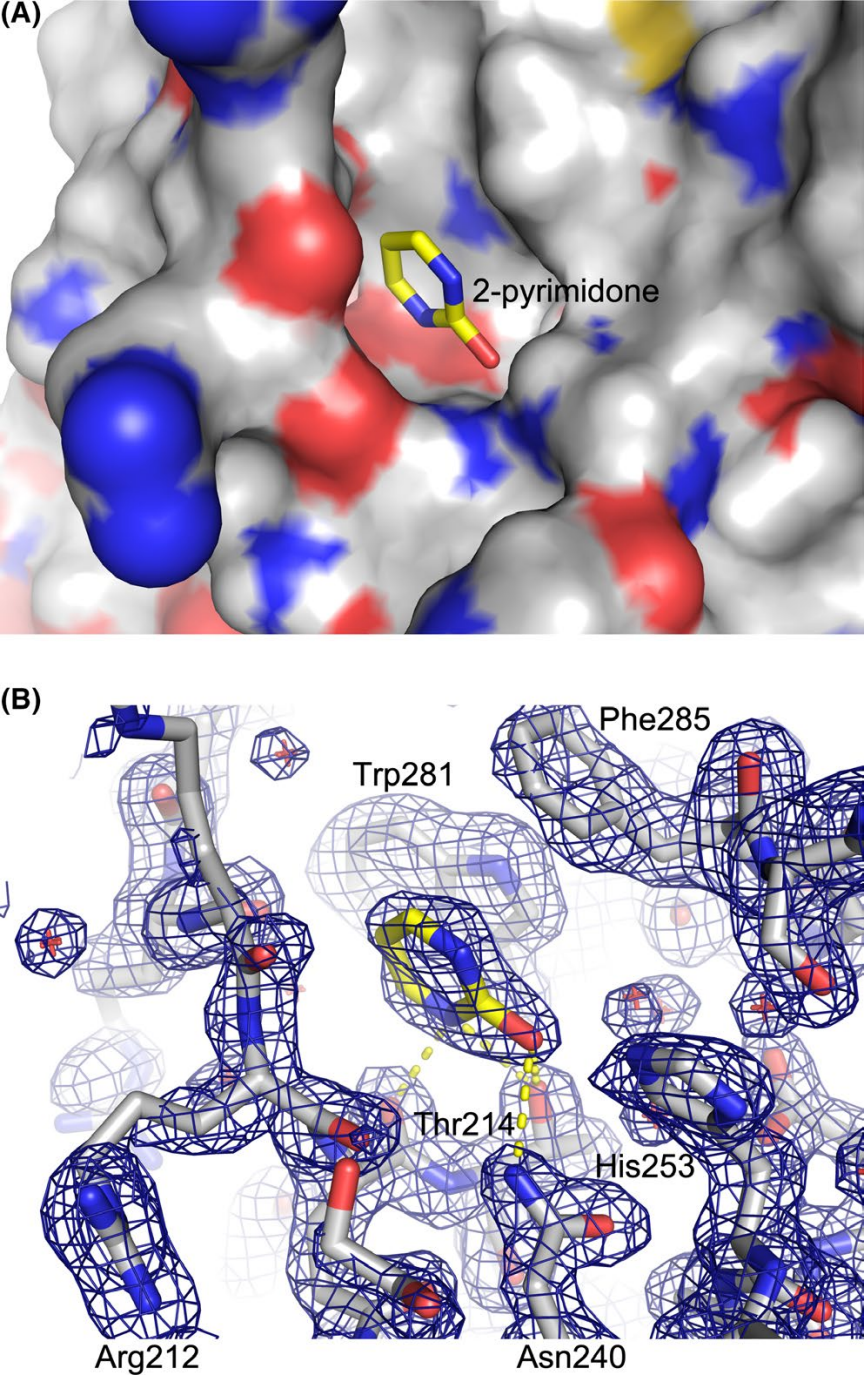
2‐pyrimidone bound in the active site. A, Solvent‐accessible surface of A3B‐GL7 with heteroatoms colored (red: oxygen; blue: nitrogen; yellow: sulfur) and 2‐pyrimidone shown as sticks. B, Protein and the bound 2‐pyrimidone both as sticks, overlaid with 2mFo‐DFc electron density in blue mesh contoured at 1.0 σ. Possible hydrogen‐bonds are indicated by yellow dotted lines

**Figure 4:**
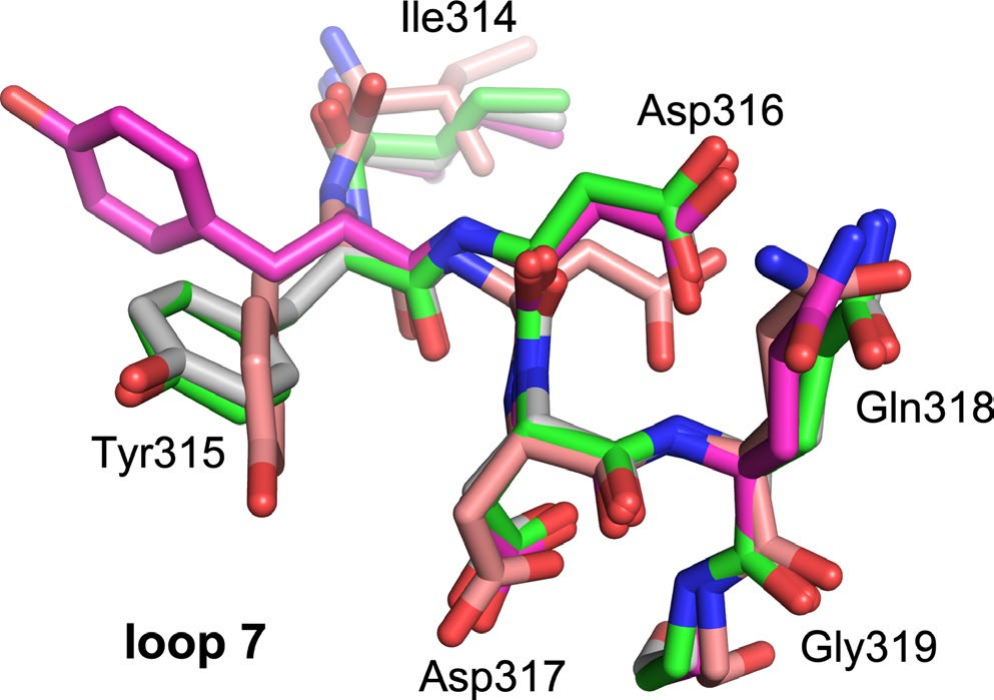
A3G loop 7 conformations. Local superposition of the loop 7 region of A3B‐GL7 and A3G structures (Figure 1A‐C and E). Coloring scheme follows that in Figure 1. Residue numbering is from native A3G

The unique conformation of the zinc‐free active site of A3B‐GL7 features a network of aromatic interactions involving Trp281, Phe285, and Tyr313 (Figures 1A,B and 2C). Phe285, which is located between the two zinc‐coordinating cysteines (Cys284/Cys289) in the amino acid sequence of A3B and is solvent‐accessible in the native A3Bctd structures,^21^, ^29^ is flipped into the active site. 2‐pyrimidone, instead of mimicking the substrate cytosine, fits in a pocket formed between this aromatic cluster and loop 1, being also stabilized by van der Waals contacts with Arg210 backbone and His253 side chain positioned co‐planarly with the pyrimidine moiety (Figure 3A). In this pocket, 2‐pyrimidone forms hydrogen bonds with the side chains of Thr214 and Asn240, and the backbone of Gln213 (Figure 3B), which are positioned similarly in the zinc‐bound native A3Bctd active site (eg 5cqi; Figures 1D and 2A). In comparison to the native A3B structure, the loop 1 of A3B‐GL7 harboring the stretch of arginines (Arg210/211/212) is shifted inward, slightly toward the active site. In contrast, the extensive rearrangement of the active site residues forces loop 7 away from the active site and closer to the α6 helix (Figure 1F). Although it is shifted by ~4 Å away from the active site, the grafted loop 7 of A3B‐GL7 adopts a similar local conformation as does loop 7 of A3G in its native environment (Figures 1E, 2B and 4). This suggests structural autonomy of A3G loop 7, which has been demonstrated to play a critical role in determining the target sequence preference.^33^, ^39^ The observation helps to explain why A3B‐GL7, in its active state, exhibits the A3G‐like (5′‐CC) target sequence preference.^21^

### 3.4 Iodide ion stabilizes a cis peptide bond in loop 5

**Figure 5:**
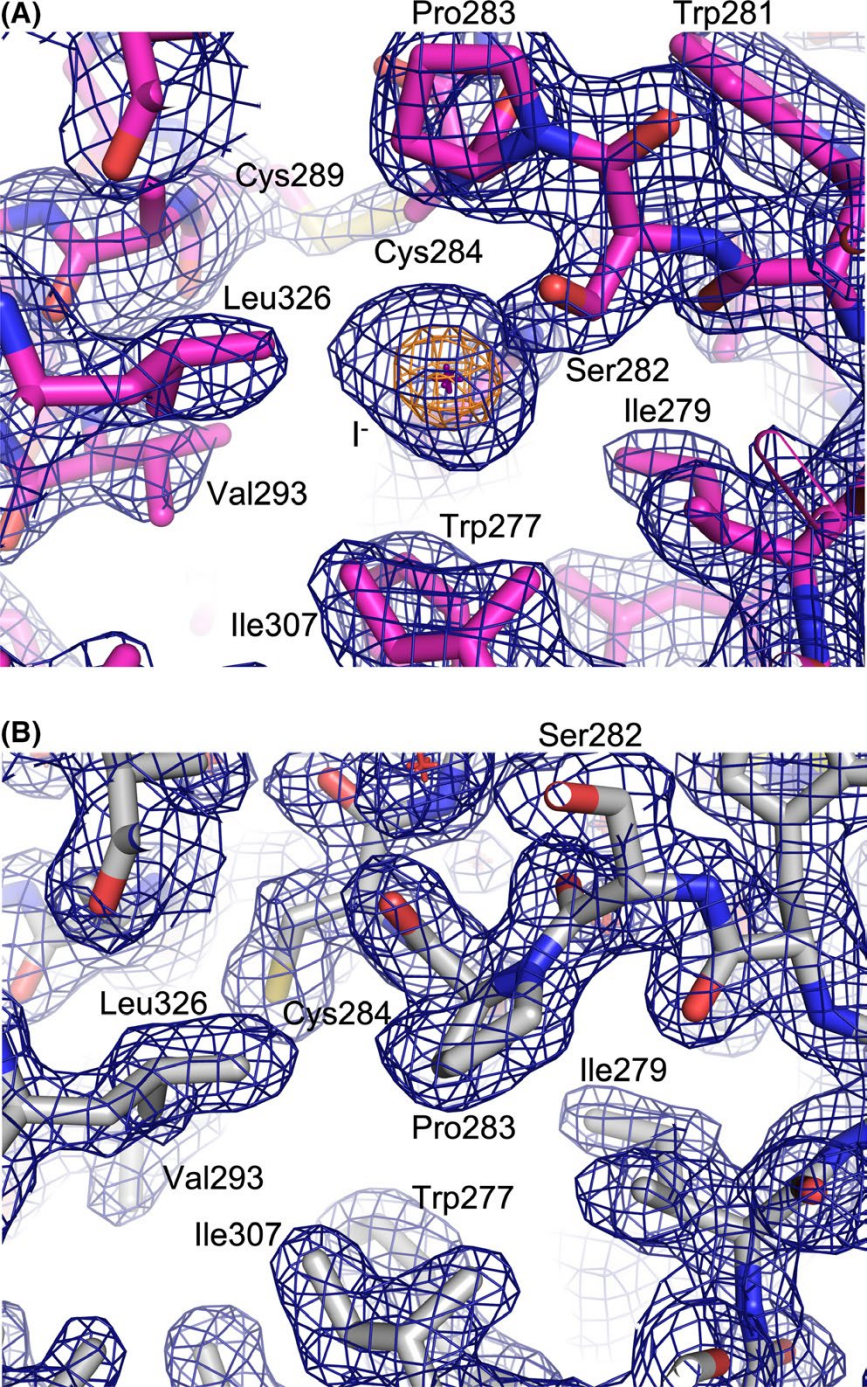
*Cis* vs. *Trans* conformations of the Ser282‐Pro283 amide bond. Zinc‐less A3B‐GL7 structure bound to an iodide ion (A, corresponding to Figure 1C) or 2‐pyrimidone (B, corresponding to Figure 1A) in stick models, overlaid with 2mFo‐DFc electron density in blue and orange mesh contoured at 1.0 and 7.5 σ, respectively. The position of iodide ion is marked by a cross. Note that Cys284 is disulfide bonded in (A) whereas it is reduced in (B), corresponding to *cis* (A) and *trans* (B) conformations of the adjacent Ser282‐Pro283 amide (peptide) bond

In addition to the two structures with and without 2‐pyrimidone as described above in similar active site conformations (Figures 1A,B and 2C), we obtained a structure of A3B‐GL7 in a crystallization condition with neutral pH and containing 45 mmol/L sodium iodide (NaI). The resulting structure refined to 1.9 Å resolution again showed a zinc‐free active site. However, in this structure loop 5 residues (Trp281‐Gly288) are further rearranged and the zinc‐coordinating cysteine residues (Cys284/289) formed a disulfide (Figure 1C). A key feature of this structure is the *cis* conformation for the Ser282‐Pro283 peptide bond (Figure 5A). This is highly unexpected, as these conserved Ser‐Pro residues adopt a more thermodynamically favorable *trans* conformation in all other A3 structures reported to date, including those of A3B‐GL7 described above (Figure 5B). In the structure obtained with NaI, we observed a strong (>15σ) spherical electron density directly adjacent to the flipped proline side chain and surrounded by hydrophobic side chains Trp277, Ile279, and Ile307, which we interpreted to be an iodide ion (Figure 5A). It is likely that an iodide ion occupying this site stabilized the distorted conformation of loop 5 and facilitated disulfide‐bond formation in the zinc‐free active site.

### 3.5 Molecular dynamics simulations reveal A3B‐GL7 active site plasticity

**Figure 6:**
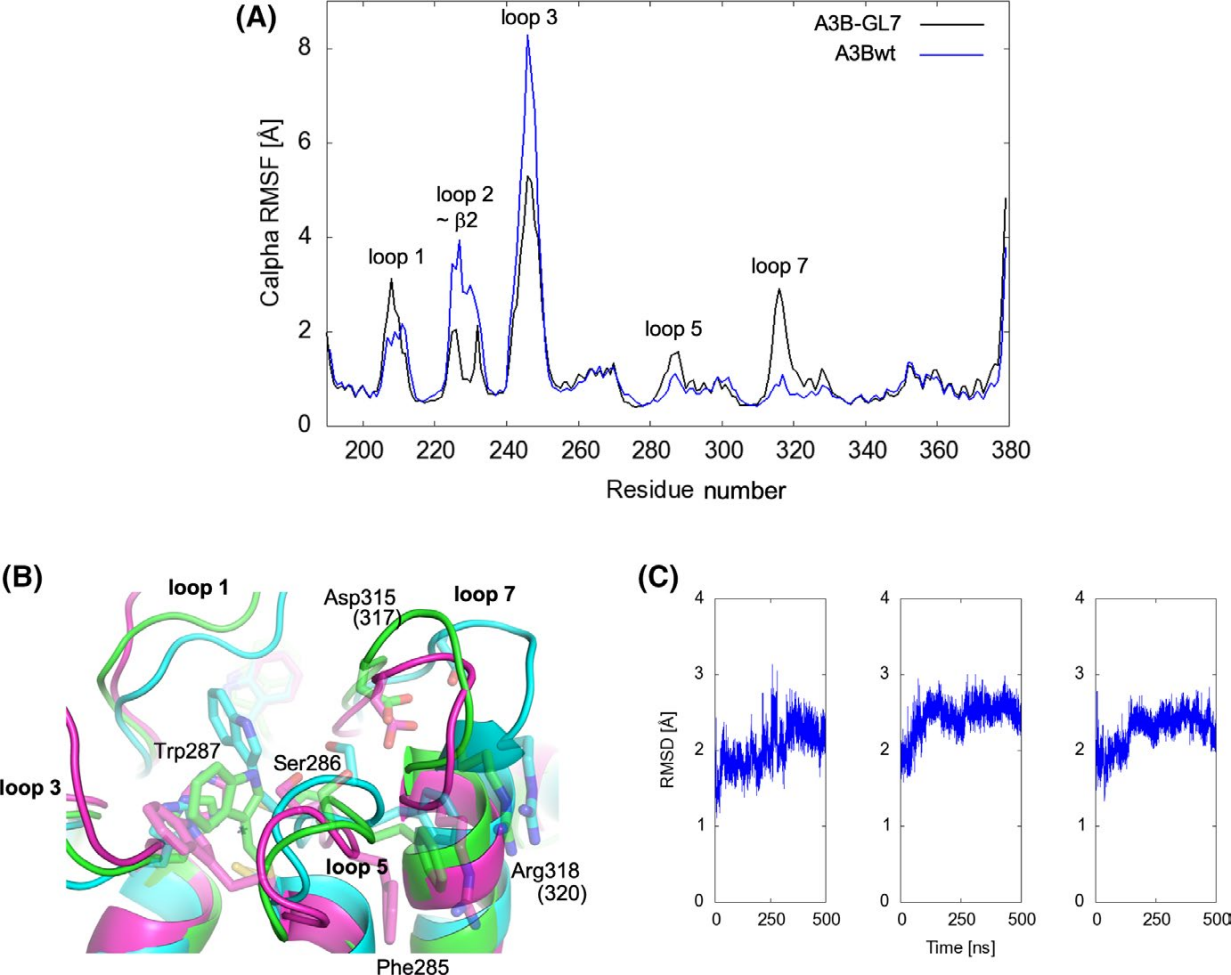
Molecular Dynamics simulations demonstrating active site plasticity. A, RMSF analysis comparing the zinc‐bound A3B_wt_‐GL7 and wild‐type A3Bctd. The A3B_wt_‐GL7 starting model was constructed by swapping the loop 7 residues of A3Bctd (similar to Figures 1D and 2A, but reverted computationally to wild‐type) with those from A3G (as in Figures 1E and 2B). B, Superposition of the final frames of three independent 500‐ns MD simulations of the zinc‐bound A3B_wt_‐GL7, showing divergence of the loops that surround the active site. Some side chains are shown as semi‐transparent sticks. C, RMSD of Cα atoms with respect to the initial frame for each system plotted to show the stability of MD simulations

To understand the molecular basis of the extensive rearrangements of A3B‐GL7 active site, we performed a series of molecular dynamics (MD) simulations. MD simulations of a computationally constructed zinc‐bound A3B_wt_‐GL7, which has the wild‐type A3B sequence restored otherwise, showed elevated dynamics compared to wild‐type A3B, not only for the grafted loop 7 but also the neighboring loops 1 and 5 (Figure 6A). In wild‐type A3B, Asp316 from loop 7 showed persistent hydrogen bonding with Phe285 and Ser286 from loop 5, as observed in the crystal structure (Figure 2A). These interactions stabilize loop 5 of A3B, which has extra amino acids between the zinc‐coordinating Cys residues compared to that of A3G (Figure 2D), in a unique conformation. In contrast, the grafted loop 7 in A3B‐GL7 does not support this native conformation of loop 5. Arg318 from loop 7 (corresponding to A3G Arg320) in the zinc‐bound A3B_wt_‐GL7 MD simulations predominantly made a cation‐π interaction with Phe285 side chain (Figure 6B), whereas it forms hydrogen bonds with loop 5 backbone in the zinc‐free A3B‐GL7 crystal structures (Figure 2C). These observations provide an explanation for the extensive active site rearrangements observed in the crystal structures of A3B‐GL7 (Figure 1A‐C), including the loss of the zinc ion.

## 4 DISCUSSION

The crystal structures of A3B‐GL7 captured in distinct inactive conformations (Figure 1A‐C and 2C), combined with MD simulation results (Figure 6), highlight remarkable plasticity of the A3B active site and identify key interactions that stabilize the native A3B active site. Of particular interest is the previously unappreciated role of loop 7 of A3B in maintaining the catalytically essential zinc ion, through stabilizing loop 5 in the conformation compatible with zinc‐coordination. Although high structural similarity between the A3B and A3G catalytic domains allowed the generation of a catalytically competent chimeric enzyme A3B‐GL7, this chimeric protein was found to lose the zinc ion more readily than A3B. This is likely to reflect a critical difference between the A3B and A3G active sites; whereas the two zinc‐coordinating cysteines of A3G (Cys288/Cys291) are part of an α‐helix (Figure 2B), the corresponding A3B residues Cys284/Cys289 are separated by additional intervening residues as part of loop 5, requiring additional hydrogen‐bonding interactions to maintain a conformation compatible with zinc‐coordination (Figure 2A). Typical zinc‐finger motifs, in which a set of 4 cysteine or histidine residues coordinate an zinc ion with tetrahedral geometry, are found in numerous proteins and often contribute to protein folding and stability.^40^, ^41^, ^42^, ^43^ In contrast, the zinc ion in the active site of A3 enzymes is coordinated by 2 cysteines and a histidine, leaving the fourth position of tetrahedral coordination available for engaging the nucleophilic water molecule. Thus, while zinc‐binding in the A3B active site is not required for domain folding, it is essential for catalytic activity and critically dependent on conformations of the surrounding protein residues.

Our studies also suggest possible means by which small molecules could inhibit A3B activity. Our results show that, while the catalytic zinc ion in APOBEC3 active sites is not essential for the domain fold, loss of the zinc ion causes dramatic rearrangement of the A3B active site. The two distinct inactive conformations that we observed, facilitated by substitution of loop 7 to destabilize the native A3B active site, allowed binding of 2‐pyrimidone or an iodide which in turn may have helped stabilize these unique conformations. Even though 2‐pyrimidone and iodide ion themselves are not potent inhibitors (Figures S1 and S2), they interact with the amino acid residues found in wild‐type A3B. We therefore envision that novel small molecules that achieve higher affinity to stabilize these non‐productive conformations might serve as chemical inhibitors for A3B, especially given the remarkable conformational plasticity of the A3B active site as demonstrated by MD simulations here, as well as in our prior studies.^28^, ^29^ In this regard, the A3B‐GL7 structures presented may contribute to the future development of potent A3B inhibitors, which we hypothesize will help to control tumor evolution and other mutational processes relevant to human diseases.

## CONFLICT OF INTEREST

RSH and DAH are co‐founders, shareholders, and consultants of ApoGen Biotechnologies Inc HA and REA are consultants for ApoGen Biotechnologies Inc REA is a co‐founder of Actavalon Inc The other authors have no competing financial interests to declare.

## AUTHOR CONTRIBUTIONS

D.A. Harki, R.E. Amaro, R.S. Harris, and H. Aihara designed the research and wrote the paper; K. Shi and S. Banerjee performed the x‐ray crystallographic analyses.
