# Supplementary material for: Active site plasticity and possible modes of chemical inhibition of the human DNA deaminase APOBEC3B
Source: FASEB Bioadv. 2019 Dec 24;2(1):49–58. doi: 10.1096/fba.2019-00068 (PMC6996314; doi:10.1096/fba.2019-00068)
Supplement: Supplementary file 1 [file FBA2-2-49-s001.pdf]

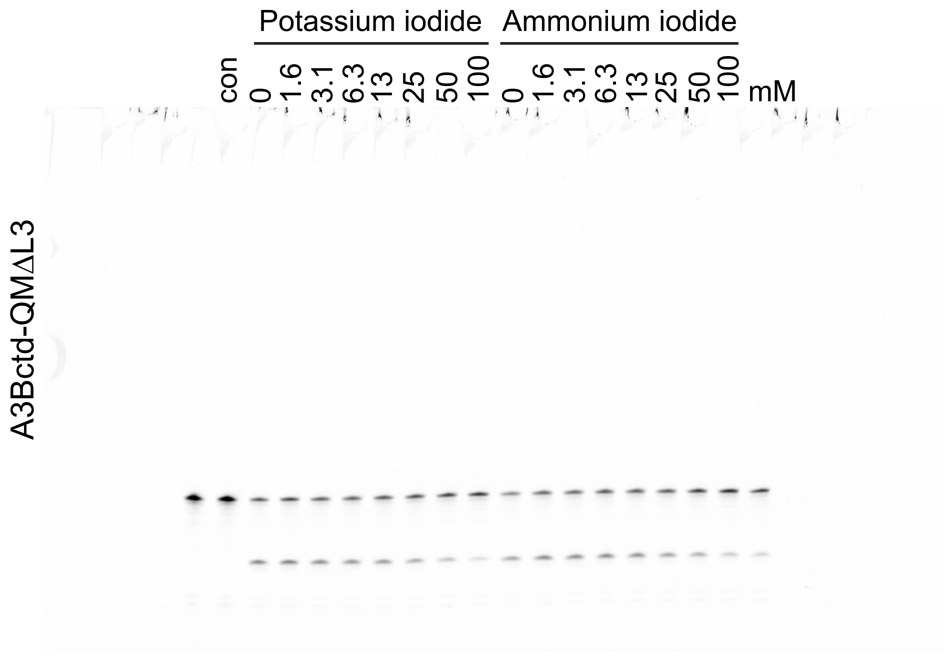
Potassium iodide
Ammonium iodide
con 0 1.6 3.1 6.3 13 25 50 100 0 1.6 3.1 6.3 13 25 50 100 mM
A3Bctd-QMΔL3

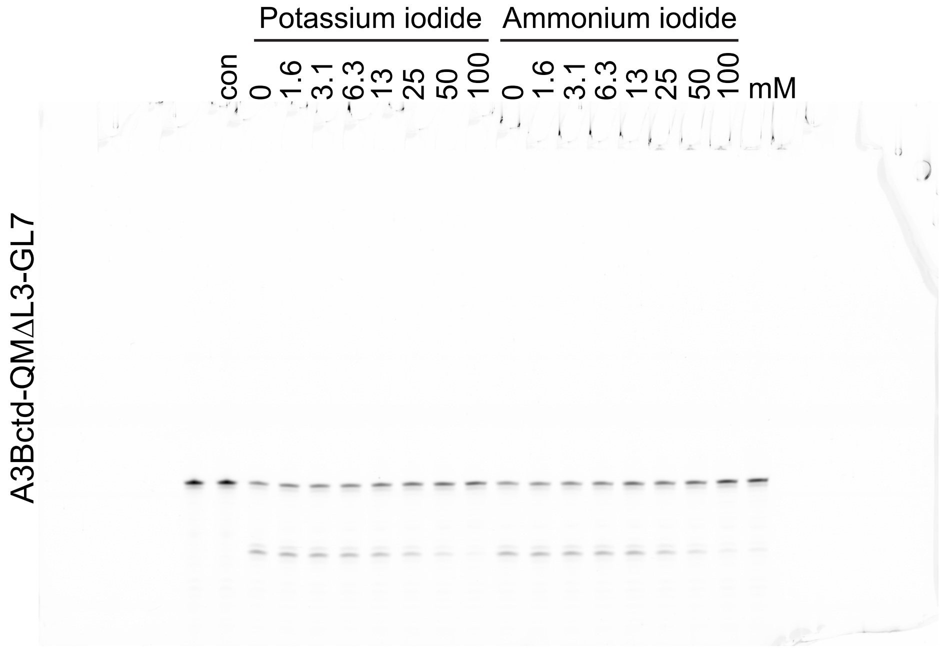
Potassium iodide
Ammonium iodide
con 0 1.6 3.1 6.3 13 25 50 100 0 1.6 3.1 6.3 13 25 50 100 mM
A3Bctd-QMΔL3-GL7

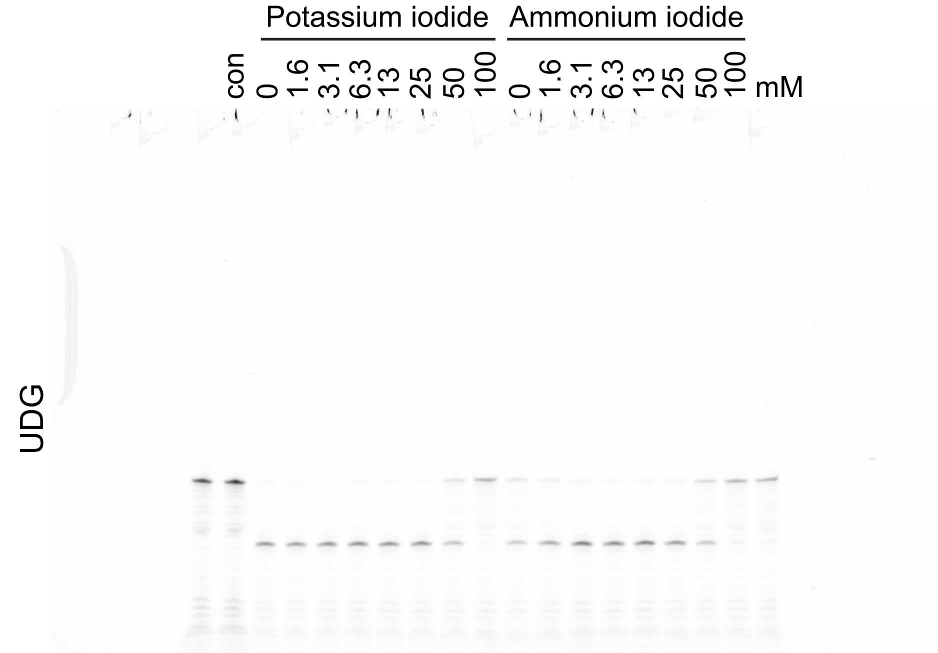
Potassium iodide
Ammonium iodide
con 0 1.6 3.1 6.3 13 25 50 100 0 1.6 3.1 6.3 13 25 50 100 mM
UDG

Supplementary Figure S1

In vitro single-stranded DNA deaminase activity of A3Bctd-QMΔL3 (top) and A3Bctd-QMΔL3-GL7 (A3B-GL7, middle) or the uracil excision activity of UDG (bottom), tested on their optimal substrates (RSH5194, RSH6700, and RSH4782, respectively) in the presence of 0-100 mM potassium iodide or ammonium iodide. Note that a higher A3B enzyme concentration and a slightly different buffer condition than those used in the experiment shown in Supplementary Figure S2 were used here. On each gel, the outside lanes are loaded with the immediately adjacent sample to reduce edge effects when running the gels.

```
RSH5194: 5’-ATTATTATTATTCAAATGGATTTATTTATTTATTTATTTATTT-fluorescein
RSH6700: 5’-ATTATTATTATCCCAATGGATTTATTTATTTATTTATTTATTT-fluorescein
RSH4782: 5’-ATTATTATTATTUTAATGGATTTATTTATTTATTTATTTATTT-fluorescein
```
