# Supplementary material for: Active site plasticity and possible modes of chemical inhibition of the human DNA deaminase APOBEC3B
Source: FASEB Bioadv. 2019 Dec 24;2(1):49–58. doi: 10.1096/fba.2019-00068 (PMC6996314; doi:10.1096/fba.2019-00068)
Supplement: Supplementary file 2 [file FBA2-2-49-s002.pdf]

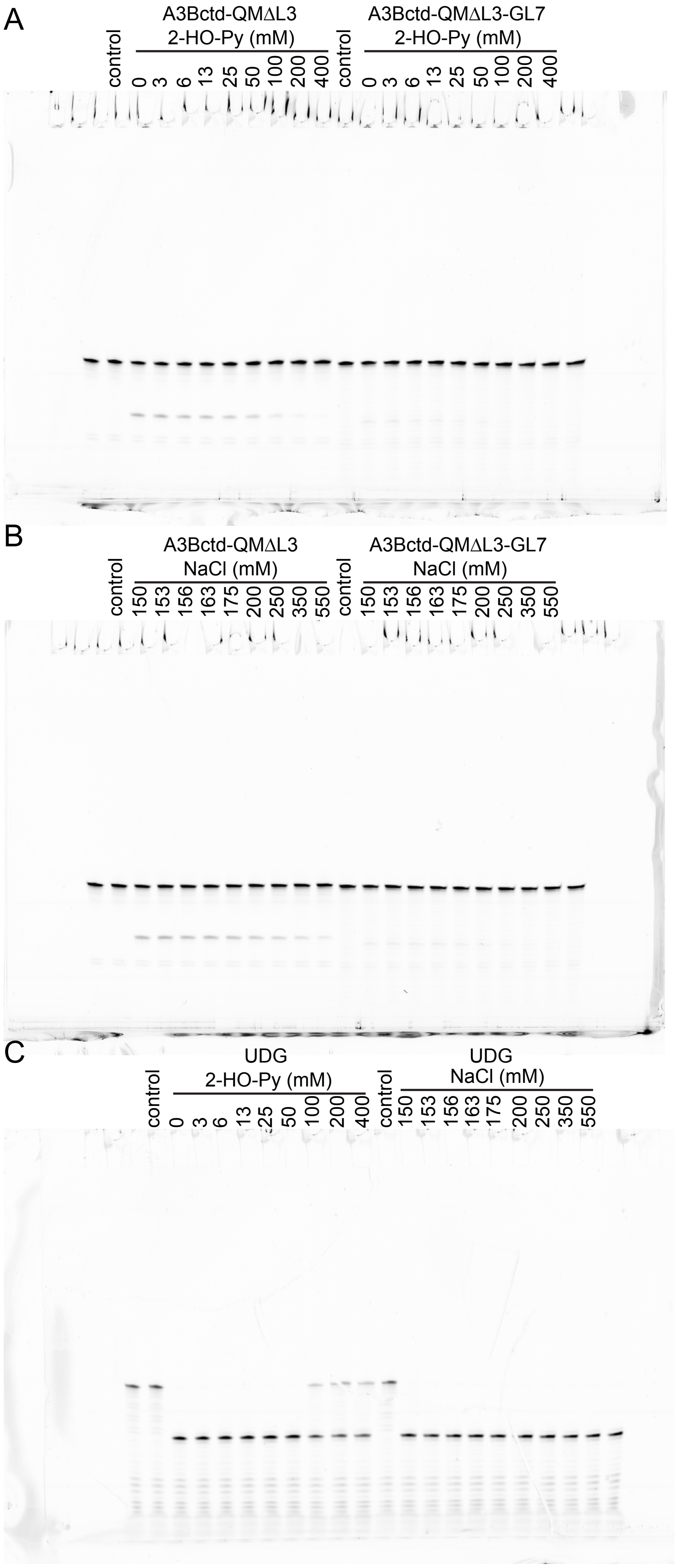
A
A3Bctd-QMΔL3
2-HO-Py (mM)
control
0 3 6 13 25 50 100 200 400
A3Bctd-QMΔL3-GL7
2-HO-Py (mM)
control
0 3 6 13 25 50 100 200 400
B
A3Bctd-QMΔL3
NaCl (mM)
control
150 153 156 163 175 200 250 350 550
A3Bctd-QMΔL3-GL7
NaCl (mM)
control
150 153 156 163 175 200 250 350 550
C
UDG
2-HO-Py (mM)
control
0 3 6 13 25 50 100 200 400
UDG
NaCl (mM)
control
150 153 156 163 175 200 250 350 550

Supplementary Figure S2

In vitro single-stranded DNA deaminase activity of A3Bctd-QMΔL3 and A3Bctd-QMΔL3-GL7 (A3B-GL7), tested on their optimal substrates (RSH5194 and RSH6700, respectively) in the presence of **A**) 2-hydroxypyrimidine from 0 to 400 mM or **B**) 0-400 mM of additional sodium chloride (150-550 mM total). **C**) In parallel with the UDG treatment of the A3B treated samples, a uracil-containing oligo (RSH4782) was treated with UDG in the presence of either 2-hydroxypyrimidine or sodium chloride to control for the possibility that 2-hydroxypyrimidine or high salt concentrations inhibit UDG. The results show that under the conditions used, high salt did not inhibit UDG but high concentrations of 2-hydroxypyrimidine did inhibit UDG. On each gel, the outside lanes are loaded with the immediately adjacent sample to reduce edge effects when running the gels.

```
RSH5194: 5'-ATTATTATTATTCAAATGGATTTATTTATTTATTTATTTATTT-fluorescein
RSH6700: 5'-ATTATTATTATCCCAATGGATTTATTTATTTATTTATTTATTT-fluorescein
RSH4782: 5'-ATTATTATTATTUTAATGGATTTATTTATTTATTTATTTATTT-fluorescein
```
