# Supplementary material for: Active site plasticity and possible modes of chemical inhibition of the human DNA deaminase APOBEC3B
Source: FASEB Bioadv. 2019 Dec 24;2(1):49–58. doi: 10.1096/fba.2019-00068 (PMC6996314; doi:10.1096/fba.2019-00068)
Supplement: Supplementary file 3 [file FBA2-2-49-s003.pdf]

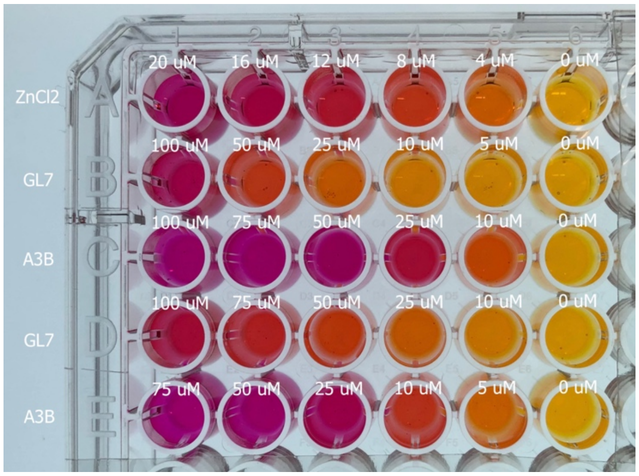

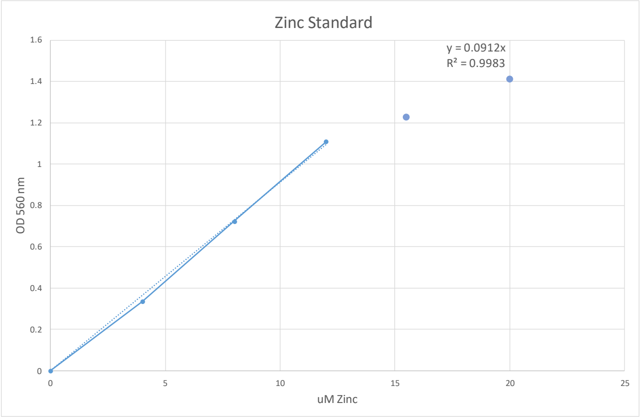

<>	1	2	3	4	5	6	7
A	1.4043	1.2395	1.1087	0.7235	0.3348	0	Zinc Standard
B	1.19	0.7044	0.346	0.1378	0.0873	-0.0029	GL7
C	1.6504	1.6555	1.6042	1.2085	0.5372	0.0051	A3B
D	1.2598	1.0028	0.7117	0.3548	0.1398	0.0025	GL7
E	1.6796	1.6185	1.3703	0.7846	0.3205	0.0035	A3B

Supplementary Figure S3

Zinc content analysis of A3Bctd-QMΔL3 (A3B) and A3Bctd-QMΔL3-GL7 (GL7). The proteins were first denatured by the addition of trichloroacetic acid, and supernatant after centrifugation was subjected to the colorimetric assay in a 96-well plate (upper left). For $ZnCl_2$ standard in the first row, final zinc concentrations of 0-12 μM after dilution with the coloring reagent showed a linear relationship with OD_{560} (upper right). The final protein concentration indicated above each well is that of the original undenatured protein (which would have been present in the final reaction mixture if it had not been pelleted) after dilution with the coloring reagent. Different batches of A3B were used for rows C and E, whereas GL7 in rows B and D were from the same prep. OD_{560} readings after background subtraction are shown in the table. Readings outside the linear range (numbers in gray) were not used in the concentration calculation. The zinc content of A3B and GL7 were estimated to be 71.7 ± 13.6 % and 15.8 ± 1.40 %, based on 3 and 8 data points within the linear range, respectively.
